# Different strategies, distinguished cooperation efficiency, and brain synchronization for couples: An fNIRS‐based hyperscanning study

**DOI:** 10.1002/brb3.1768

**Published:** 2020-07-25

**Authors:** Yun Tang, Xin Liu, Chenbo Wang, Miao Cao, Shining Deng, Xiujuan Du, Yuan Dai, Shujie Geng, Yun Fan, Lijuan Cui, Fei Li

**Affiliations:** ^1^ School of Psychology and Cognitive Science East China Normal University Shanghai China; ^2^ Developmental and Behavioral Pediatric Department & Child Primary Care Department Brain and Behavioral Research Unit of Shanghai Institute for Pediatric Research MOE‐Shanghai Key Laboratory for Children’s Environmental Health Xinhua Hospital Shanghai Jiao Tong University School of Medicine Shanghai China; ^3^ Institute of Science and Technology for Brain‐Inspired Intelligence Fudan University Shanghai China; ^4^ Key Laboratory of Computational Neuroscience and Brain‐Inspired Intelligence Fudan University Ministry of Education Shanghai China

**Keywords:** cooperation coefficient, cooperation keystroke task, functional near‐infrared spectroscopy, interpersonal brain synchronization, strategy

## Abstract

**Introduction:**

Individuals may employ different strategies when cooperating with others. For example, when two participants are asked to press buttons simultaneously, they may press the buttons as quickly as possible (immediate response strategy) or press them in a delayed pattern (delayed response strategy). Despite recognition of interpersonal brain synchronization (IBS) as a fundamental neural mechanism of cooperation, it remains unclear how various strategies influence cooperative behavior and its neural activities.

**Methods:**

To address this issue, 43 married couples were recruited to complete a button‐press cooperative task, during which IBS was recorded by functional near‐infrared spectroscopy hyperscanning.

**Results:**

Behavioral results showed that couples who adopted a delayed response strategy performed better than those who adopted an immediate response strategy and those without any obvious strategy, and a new measure (cooperation coefficient) was used to index the level of cooperation. In addition, stronger IBS in the right frontal cortex was observed in the delayed response condition. The greater couples’ perceived parenting stress, the more likely they were to perform well in tasks and the stronger their brain synchronization, since they tended to choose the delayed response strategy.

**Conclusion:**

The delayed response strategy may better unify dyad partners’ response modes, trigger synchronized psychological processes, and enable their brains to become synchronized. The study extends understanding of cooperation by comparing the contributions of different strategies underlying cooperative behavior with corresponding neural evidence.

## Graphical abstract

Pressing keys in a delayed pattern leads to better performance and more synchronized brain activation than pressing them as quickly as possible. The delayed response strategy may better unify a dyad’s response modes: it enables both partners to react according to the established rhythm, triggers synchronized psychological processes, such as predicting each other’s keystrokes and deciding when to press, and enables their brains to become synchronized. A cooperation coefficient that can better describe (compared to the winning ratio) the correlation between a dyad’s behavioral performance and the synchronization of the respective brains.

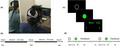

## 1 INTRODUCTION

In recent years, an fNIRS‐based hyperscanning technique has been widely used to obtain the indicator interpersonal brain synchronization (IBS), or interpersonal neuronal synchronization (INS), in various social situations. The term describes the synchronization degree of changes in the activation of specific brain areas for two (or more) partners in social activities or common tasks. Several studies using fNIRS‐based hyperscanning techniques have shown that IBS represents the quality of interpersonal communication to a large extent. For example, it can predict face‐to‐face conversation (Jiang et al., 2012), which differs from other communication modes, and can completely mediate the influence of behavioral synchronization on two partners’ prosociality toward each other (Hu, Hu, Li, Pan, & Cheng, 2017). It has also been found to completely mediate the relationship between parents’ emotional regulation ability and that of their children (Reindl, Gerloff, Scharke, & Konrad, 2018).

Many studies have used this technique to measure IBS in cooperative keystroke tasks (Cui, Bryant, & Reiss, 2012), and various studies have basically reached the same conclusion that IBS can significantly predict task performance (Baker et al., 2016; Cui et al., 2012; Pan, Cheng, Zhang, Li, & Hu, 2017; Reindl et al., 2018; Wang, Zhang, et al., 2019; Wang, Han, et al., 2019). However, based on various experimental designs, these studies have different explanations for the variance in participants’ task performance and IBS. For example, external stimuli, such as capsaicin‐induced pain, were found to motivate participants to seek social support, considerably improving their initially poor collaboration (i.e., dyad partners’ key‐press response patterns became more consistent), and triggering significant IBS in the left lateral prefrontal cortex and right parietal cortex (Wang, Zhang, et al., 2019).

Another important factor probably influencing task performance and IBS is the dyad's gender composition. Baker et al. (2016) found that dyads with at least one male achieved significantly better performance than those with two females. This may be because males tend to adopt action modes based on personal perception and mentalization, which are more conducive to cooperative performance, while females rely more on behavior‐centered social perceptions, whereby they simply use the other partner's actions in the task to determine their own response. In Baker et al.’s (2016) cooperative tasks study, the distinction between the two modes is mainly based on differences in the brain regions where IBS is produced by dyads with different gender compositions: male–male dyads produced significant IBS in the frontal pole, female–female dyads produced IBS in the temporal cortex, and mixed‐sex dyads failed to produce significant IBS in the whole right frontal‐parietal region. However, Pan et al.’s (2017) findings for mixed‐sex dyads were more nuanced: compared to mixed‐sex dyads comprising either friends or strangers in college, college student lovers performed better in cooperative tasks, and with more significant IBS than others. The reason may be that the cooperation between lovers involves more emotions, which stimulate the motivation, especially in boyfriends, to achieve better task performance. Consequently, boyfriends adjusted their response patterns to match the response rhythm of their girlfriends. Taken together, both task performance and IBS were related to the consistency between dyad partners’ responses, which might result from social support from at least one partner to the other. This leads to an interesting question: Is there a similar motive for a couple who have already been married for years (perhaps most of them with some parenting stress), where one is the dominant partner and cooperates actively with the other? If not, is there any possibility for the couple to form a strategy to unify their response rhythm? This is the area investigated by the present study.

**Figure 1:**
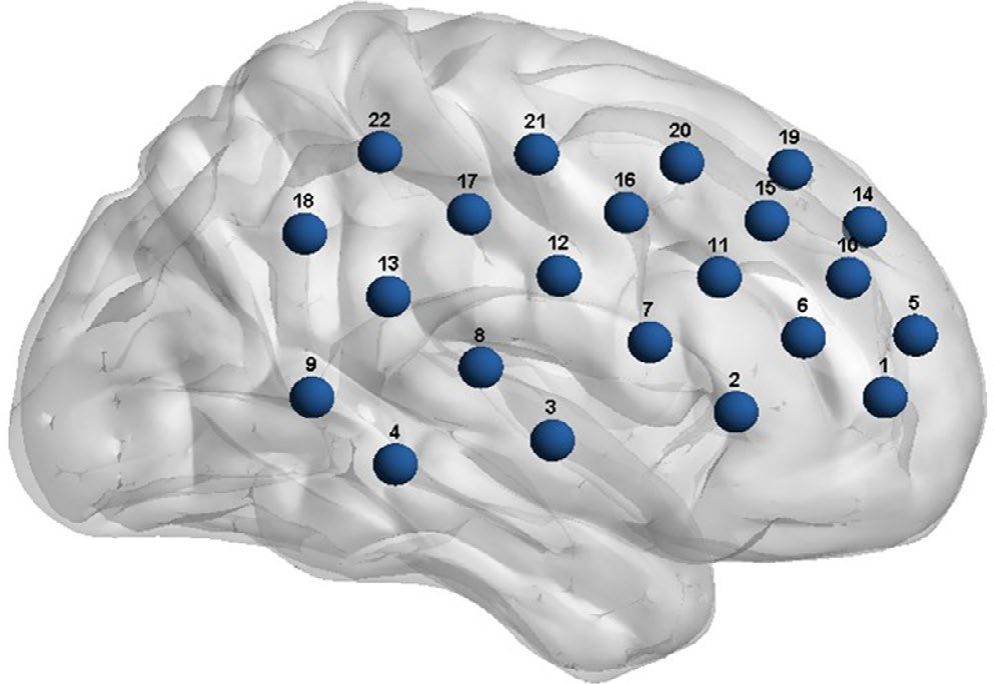
Measuring channels’ positions. The selected region of interest (ROI) was the right frontal‐parietal region

Existing studies based on the cooperative keystroke task and fNIRS‐based hyperscanning have found that IBS is mainly produced in bilateral frontoparietal cortices (Baker et al., 2016; Cui et al., 2012; Hu et al., 2017; Pan et al., 2017; Reindl et al., 2018; Wang, Han, et al., 2019), with the specific locations of IBS differing in frontal cortices; one study also found IBS in the temporal cortex (Baker et al., 2016). Pan et al. (2017) recruited lovers (the closest match to this study) and found IBS in their right frontal cortex. Previous studies have found that the right frontal‐parietal cortex is associated with interpersonal cooperation and interaction (Decety, Jackson, Sommerville, Chaminade, & Meltzoff, 2004). In addition, a right‐lateralized frontoparietal mirror‐neuron network has been shown to be associated with social understanding, for example, understanding others’ emotions and behaviors (Gallese, Keysers, & Rizzolatti, 2004; Iacoboni et al., 2005), and is considered to be the basis for bridging the self‐others gap in the interaction process (Uddin, Iacoboni, Lange, & Keenan, 2007). Therefore, based on previous studies’ results, the selected region of interest (ROI) in this study was the right frontal‐parietal region, as shown in Figure 1.

Another common feature of the above studies on the cooperative keystroke task (in which two players must press designated keys as quickly as possible when an on‐screen signal appears) is that task performance (the ratio of the number of winning trials to the total number of trials, that is, the winning ratio (WR)) is used to indicate the cooperation level. This approach seems logical but also has some shortcomings.

First, the nature of the task produces some ambiguity in WR. Winning in a single trial depends not only on the difference in response times between dyad partners but also on the threshold value of the trial. In a single trial, if the response time difference (RTD) is less than or equal to the threshold value, the dyad wins one point; otherwise, it loses one. However, labels such as “win” or “loss” do not reflect the specific difference between RTD and the threshold value. Obviously, this is a loss for determining the cooperation level in dyads and for subsequent statistical analysis. Second, several studies have suggested that IBS can predict task performance mainly because there are significant differences in both performance and IBS among different experimental groups. However, the correlation between task performance and IBS has not been directly tested. In theory, there is a possibility of overestimating or underestimating this predictive relationship.

Third, on this basis, interpretation of the correlation between performance and IBS in brain regions is of questionable validity. Therefore, we believe that the indicator used to represent a dyad's cooperation level in the cooperative keystroke task should be reconstructed, based on two key factors that are more objective and reflect the characteristics of dyad partners’ responses: RTD and threshold.

In this study, an fNIRS‐based hyperscanning technique was used to measure the effect of a cooperative strategy on task performance and IBS, and a cooperation coefficient (CC) was constructed based on dyad partners’ RTD and threshold. We hypothesize that 1) the strategy that is most conducive to unifying partners’ response rhythm will enable them to achieve a high level of cooperation; 2) CC can represent dyad partners’ cooperation level and significantly predict their task performance and IBS.

## 2 METHOD

### 2.1 Participants

From March 2018 to March 2019, we recruited parents of 43 children (aged from 5.5 to 14, 8.01 ± 2.15) who sought developmental suggestions from the Developmental and Behavioral Pediatric Department & Child Primary Care Department of Xinhua Hospital, affiliated with Shanghai Jiao Tong University School of Medicine. The fathers’ age ranged from 31 to 50 years old (38.79 ± 4.6), and the mothers’ age ranged from 30 to 43 years old (36.39 ± 3.45). None of the parents had any kind of mental disorder. Around two‐thirds of the children were diagnosed as having autism spectrum disorder (ASD) or attention deficit hyperactivity disorder (ADHD); the other one‐third are typically developmental according to the results of regular physical checkups.

The study was approved by the Ethical Committee at Xinhua Hospital and conducted in accordance with the relevant guidelines and regulations. Written informed consent was obtained from all participants.

### 2.2 Experimental tasks and procedures

**Figure 2:**
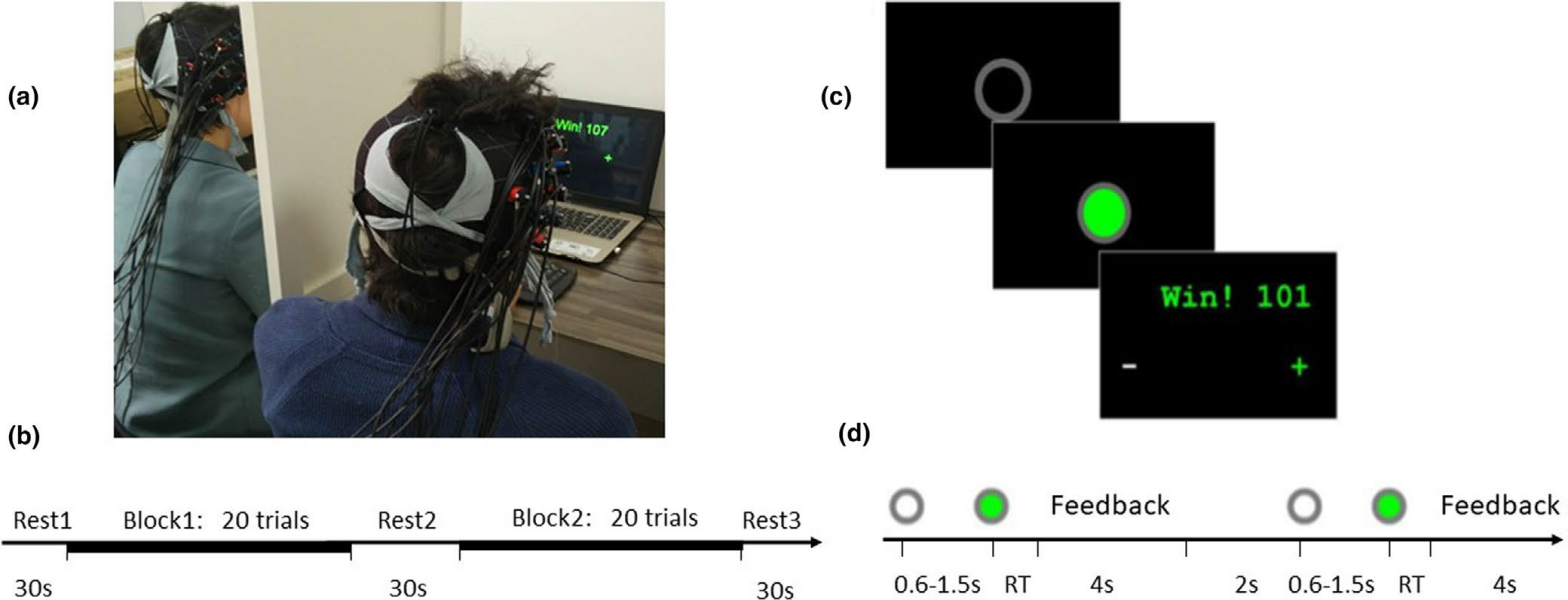
Experimental procedure and task flow of each trial. (a) Experimental setup. (b) Task design. There were two task blocks, each comprising 20 trials. (c and d) Trial design, depicting events and time flow in a trial

The experimental tasks were consistent with those of Cui et al. (2012) and others (e.g., Pan et al., 2017). Dyad partners sat side‐by‐side in front of a shared computer monitor, separated by a baffle (Figure 2a). Task procedures included rest 1 (30 s), task block 1 (~150 s), rest 2 (30 s), task block 2 (150 s), and rest 3 (30 s) (Figure 2b). For each task block, participants needed to complete 20 trials. In each trial (Figure 2c/d), a hollow gray circle (0.6–1.5 s) appeared first, followed by a green signal. When seeing the signal, the participants pressed the keys as soon as possible. The left participant (Participant 1) pressed the “z” key on the keyboard, and the right participant (Participant 2) pressed the “/” key on the keyboard. When RTD between the two partners was less than the threshold value, the dyad won one point; otherwise, it lost one point. Then, the system gave feedback to show the current cumulative score and the response times of both partners (“–” means the corresponding subject's response time was longer, and “+” means it was shorter), so that the dyad could adjust in the next trial. The calculation formula for the threshold was *T* = (RT1 + RT2)/8. RT1 and RT2 were the response times of Participants 1 and 2, respectively. A parameter of 1/8 was selected to maintain a reasonable level of difficulty (Baker et al., 2016; Cui et al., 2012; Pan et al., 2017).

First, each couple (as a cooperative dyad) practiced 20 trials, during which partners could communicate with each other on how to coordinate in this task, so as to form a cooperative strategy. The researchers did not give any guidance or hints, and told participants that they would not be allowed to communicate with each other during the formal task. According to on‐the‐spot observation of dyads’ practices, researchers formed a preliminary judgment on whether the participants had formed a consistent cooperative strategy and, if so, what kind. Next, a formal cooperative keystroke task was conducted. At this point, dyad partners could no longer have any form of communication, and a Hitachi ETG‐4000 fNIRS device was used to record changes in the blood oxygen level in specific brain regions of dyad partners (Figure 2a). After the formal task was completed, researchers orally confirmed and recorded the participants’ strategy. Video recordings were made of each experiment.

### 2.3 fNIRS acquisition

The concentration changes of oxyhemoglobin (oxy‐Hb) and deoxyhemoglobin (deoxy‐Hb) were measured with the ETG‐4000 at a sampling frequency of 10 Hz. We used two “3 × 5” holders provided by Hitachi, one for each partner. In each holder, eight transmitters and seven detectors were placed alternately, forming 22 measurement channels, covering a limited area of the brain. This required us to accurately determine the ROI most likely to produce IBS in this task. Otherwise, we would lose the opportunity to detect the generated IBS.

### 2.4 Scales and subjective evaluation measurements

At the end of the experiment, the parents were asked to complete two scales: the Parenting Stress Index Short Form (PSI/SF) and the Dyadic Adjustment Scale (DAS). The PSI/SF has three dimensions: parenting stress, parent–child dysfunctional interaction, and difficult child; it was compiled by Abidin and translated by Wenxiang (1995). The DAS includes four subscales designed to assess marital satisfaction, marital harmony, marital cohesion, and emotional expression (Spanier, 1976). After the experiment, we also collected each participant's subjective evaluations regarding the experiment, covering: their shared intention, performance satisfaction, in‐task cooperation degree, in‐task concentration degree, and feeling of pleasantness. Items of shared intention included: “I shared the same mind with my partner in the task”; items of satisfaction included: “I am satisfied with my performance”; items of cooperation included: “We cooperated well in this task”; items of concentration included: “I was very engaged in the task”; and items of pleasantness feeling included: “I had a good time in the task.” Participants rated each item on a 5‐point Likert scale, ranging from 1 (strongly disagree) to 5 (strongly agree). No discussion between partners was allowed during the rating process.

### 2.5 Data analysis

#### 2.5.1 Strategic classification

At the end of the formal task, researchers asked dyad partners how they cooperated. If participants’ answers clearly reflected that they allowed a certain delay between seeing the signal and pressing keys, the “delayed response” strategy was recorded (the delays of different dyads varied, with the silent count usually from one to three); if their answers conveyed that they immediately pressed keys after seeing the signal, the “immediate response” strategy was recorded. In addition, a small number of dyads failed to form a consistent cooperation strategy in the practice stage: in some cases, one parent did not communicate either at all or effectively with the other in the practice stage; in other cases, there was unwillingness to cooperate, which made it impossible to form an effective cooperation strategy. There were also cases in which one partner advocated delay while the other preferred an immediate response, and cases in which both sides agreed on delay but diverged on its length (one thinking it should equal the time from cue to signal presentation, which varies between 0.6–1.5 s (Figure 2d), and the other thinking it should be fixed (such as counting to two or three) from the presentation of the stimulus). In all of these cases, the couples were at a standstill in choosing strategies and failed to reach agreement during the practice stage; we recorded them as having “no obvious strategy.” On this basis, we used ANOVA to test whether the average response times of the three groups reflected different strategy choices.

#### 2.5.2 Task performance

ANOVA was used to compare the performance (WR) of different strategy groups in the task.

#### 2.5.3 IBS

The fNIRS data were preprocessed using Homer2 (MGH Martinos Center for Biomedical Imaging, Boston, MA, USA), a MATLAB (The MathWorks, Inc., Natick, MA, USA) toolbox. The specific procedure is as follows. First, channels with low signal‐to‐noise ratio (i.e., Msignal/SDsignal < 2) were detected and treated as missing data. Second, motion artifacts were detected and corrected. However, the signals were not band‐pass filtered because a wavelet processing method was used for further analysis. Finally, the preprocessed data were transformed to oxy‐Hb and deoxy‐Hb concentrations according to the modified Beer–Lambert Law.

Oxy‐Hb signals are most sensitive to cerebral blood flow changes during fNIRS measurements (Cui et al., 2012; Hoshi, 2007), so we focused on the oxy‐Hb time series. For each channel of each pair of participants in the cooperative experiment, we have two oxy‐Hb time series (for example, oxy‐Hb in channel 5 of a dyad). Wavelet Transform Coherence (WTC) generates a two‐dimensional coherence map from the analysis of these two time series. Then, we determined the frequency band of task occurrence between 3.2 and 12.8 s, consistent with previous studies (e.g., Cui et al., 2012; Pan et al., 2017). We calculated the average coherence of the two task blocks and the interval between them. IBS is defined as the average coherence value of two task blocks minus the average coherence value in the interval. Then, we tested the IBS of each channel in each strategy group with a single sample *t* test and analyzed the variance between groups with an ANOVA (before this analysis, the consistency values were converted to Fisher Z‐statistics; Chang & Glover, 2010).

#### 2.5.4 Directional coupling

Next, we used Granger causality analysis (GCA) to estimate the direction of synchronization for channels that exhibited significance (Pan et al., 2017; Zhang, Liu, Pelowski, & Yu, 2017). GCA uses a vector autoregressive model to measure causality between time series in brain data. The G‐causality of both directions (from mother to father and from father to mother) was calculated. Then, one‐sample *t* tests were used to determine whether the G‐causality in each direction were significant, and paired‐samples *t* tests to compare the differences between the two directions.

#### 2.5.5 Relationship between subjective measurements, task performance, and IBS

Pearson correlation analyses were used to test whether there were significant correlations among task performance, IBS, and related subjective measurements.

## 3 RESULTS

### 3.1 Behavioral performance

#### 3.1.1 Cooperative strategies between couples and their task performance

**Figure 3:**
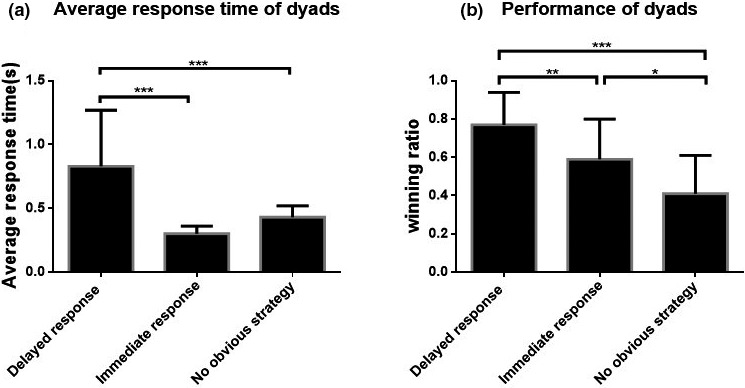
The mean response time and performance (WR) of dyads. (a) A significant difference was found in the response times of couples with different cooperative strategies (*F*(2,40) = 15.83, *p* < .001, $\eta_{p}^{2}$ = 0.442). (b) There was also a significant difference in WR between different groups (*F*(2,40) = 11.77, *p* < .001, $\eta_{p}^{2}$ = 0.370). *p < .05, **p < .01, ***p < .001

Through videos and text recordings of the experiment and conversations between the researchers and participants, 17 of the 43 couples were identified as adopting the delayed response strategy in the practice phase and then in the formal experiment; 16 couples adopted the immediate response strategy; and 10 couples failed to form a consistent and effective cooperative strategy. A significant difference was found in the response times of couples with different cooperative strategies. As shown in Figure 3a, the average response time was 0.83 s (±0.44 s) in the delayed response group, 0.30 s (±0.06 s) in the immediate response group, and 0.43 s (±0.09 s) in the no obvious strategy group (*F*(2,40) = 15.83, *p* < .001, $\eta_{p}^{2}$ = 0.442). The results also confirmed our identifications of couples’ strategic choices. Different strategy choices also determined task performance. As shown in Figure 3b, the average task WR was 0.77 (±0.17) for couples in the delayed response group, 0.59 (±0.21) for the immediate response group, and 0.41 (±0.20) for the no obvious strategy group (*F*(2,40) = 11.77, *p* < .001, $\eta_{p}^{2}$ = 0.370). This result is consistent with our first hypothesis, namely that the strategy most conducive to unifying partners’ response rhythm will enable them to achieve a high level of cooperation.

Figure 3a shows great variation in the dyads’ average response time, attributable to differences in the delay lengths agreed in the dyads. They may have silently counted to one, two, or even three after seeing the signal. The immediate response strategy group had almost no delay, so the variation in response time was very small. Variation in the no obvious strategy group fell between that of the other two groups.

#### 3.1.2 Reasons for “delay” winning

**Figure 4:**
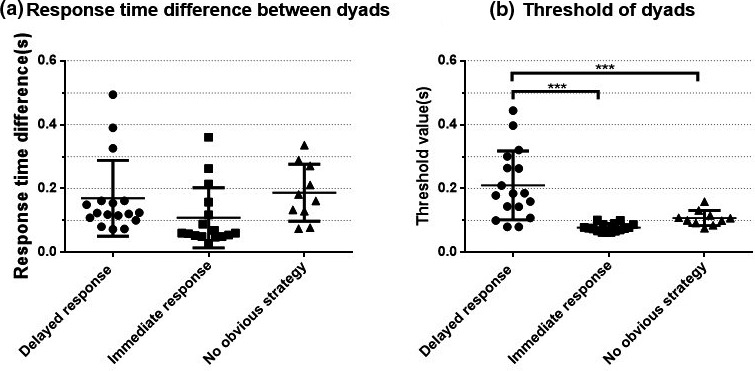
RTDs and thresholds between dyads in tasks. (a) There was no significant difference in RTDs across groups (*F*(2,40) = 2.21, *p* = .12, $\eta_{p}^{2}$ = 0.10). (b) A significant difference was found in thresholds across groups (*F*(2,40) = 15.97, *p* < .001, $\eta_{p}^{2}$ = 0.444). *p < .05, **p < .01, ***p < .001

The formula for calculating the threshold value of the RTD between dyads is (RT1 + RT2)/8, which the participants did not know. This means that the longer the RTs of the two partners in each trial (within 4 s), the greater the threshold value, and the higher the fault tolerance rate. Was the higher WR of the delayed response strategy group due to the higher threshold value or the smaller RTD between the two partners? We used ANOVA to test whether there were significant differences in RTDs and thresholds across the three groups. The results revealed no significant difference in RTDs across groups (*F*(2,40) = 2.21, *p* = .12, $\eta_{p}^{2}$ = 0.10; see Figure 4a), but a significant difference in thresholds across groups (*F*(2,40) = 15.97, *p* < .001, $\eta_{p}^{2}$ = 0.444; see Figure 4b). Post hoc analysis showed that the threshold of the delayed response group was significantly higher than that of the immediate response group (*p* < .001) and the no obvious strategy group (*p* < .001). This shows that the delayed response group performed better because these dyads’ strategy yielded a more relaxed response time threshold, although participants did not know this at the time. Figure 4b shows that the distribution of dyads’ mean threshold was very concentrated in the immediate response group but very wide in the delayed response group, due to the different delay time in the various dyads. The mean threshold distribution of the no obvious strategy group falls between those of the other two groups. As shown in Figure 4a, the mean RTD of the delayed response group was lower than its threshold mean; in contrast, the mean RTDs of the other two groups were larger than their respective threshold means. Also, most dyads in the delayed response group (14 of 17 pairs) had lower RTD than the intra‐group mean; only three pairs of partners had higher RTD, which indicates that the delayed response strategy helped dyads to achieve better task performance in general. However, the higher cooperation level required between the two partners meant that task difficulty was higher. Without good implementation, dyads cannot achieve the desired performance.

#### 3.1.3 Cooperation coefficient

**Figure 5:**
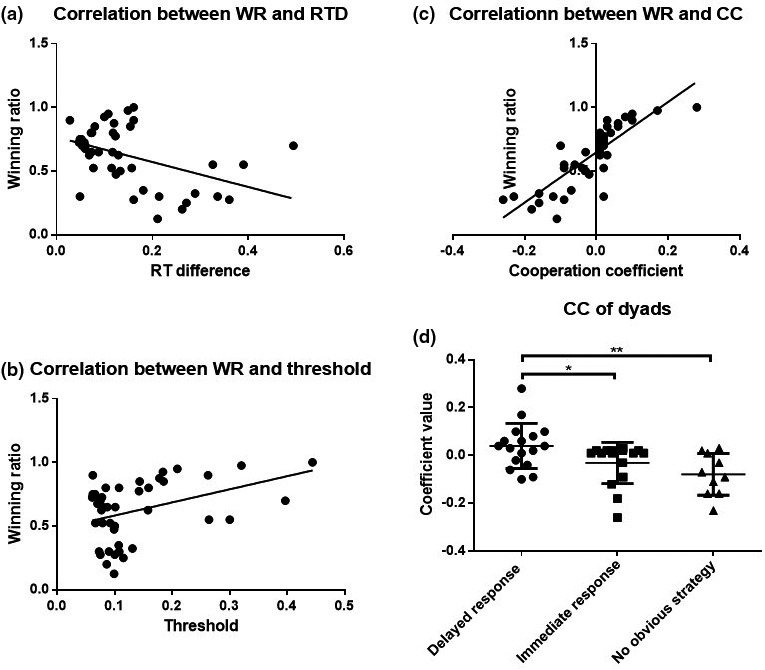
Differences in cooperation coefficients across groups and the correlation of WR with RTD, threshold, and CC (WR = winning ratio, RTD = Response time difference, CC = cooperation coefficient). (a) The correlation coefficient between RTD and WR was *r* = −.44 (*p* = .003). (b) The correlation coefficient between threshold value and WR was *r* = .398 (*p* = .008). (c) CC was more effective than WR in predicting participants’ task performance (*r* = .838, *p* < .001). (d) There were significant differences among the participants with different strategies (*F*(2,40) = 6.04, *p* < .005, $\eta_{p}^{2}$ = 0.232). *p < .05, **p < .01, ***p < .001

So far, we have confirmed there were two key factors involved in winning the cooperative tasks. One was the RTD between dyad partners: the smaller the RTD, the more likely the dyad was to win (the correlation coefficient between RTD and WR was *r* = −.44, *p* = .003; see Figure 5a). The other was the threshold value: the larger the threshold value, the more likely the dyad was to win (the correlation coefficient between threshold value and WR was *r* = .398, *p* = .008; see Figure 5b). Combining these two factors reveals the level of cooperation between partners. Accordingly, we used the results of subtracting the average RTD from the average threshold value of partners as the “cooperation coefficient” (CC): the larger CC, the higher the level of cooperation. Through analysis comparing the independent prediction of these two factors, we found that CC was more effective than WR in predicting participants’ task performance (*r* = .838, *p* < .001; see Figure 5c), and there were significant differences among the participants with different strategies (*F*(2,40) = 6.04, *p* < .005, $\eta_{p}^{2}$ = 0.232; see Figure 5d), which is consistent with our second hypothesis, namely that CC can represent dyad partners’ cooperation level and significantly predict their task performance.

### 3.2 IBS

**Figure 6:**
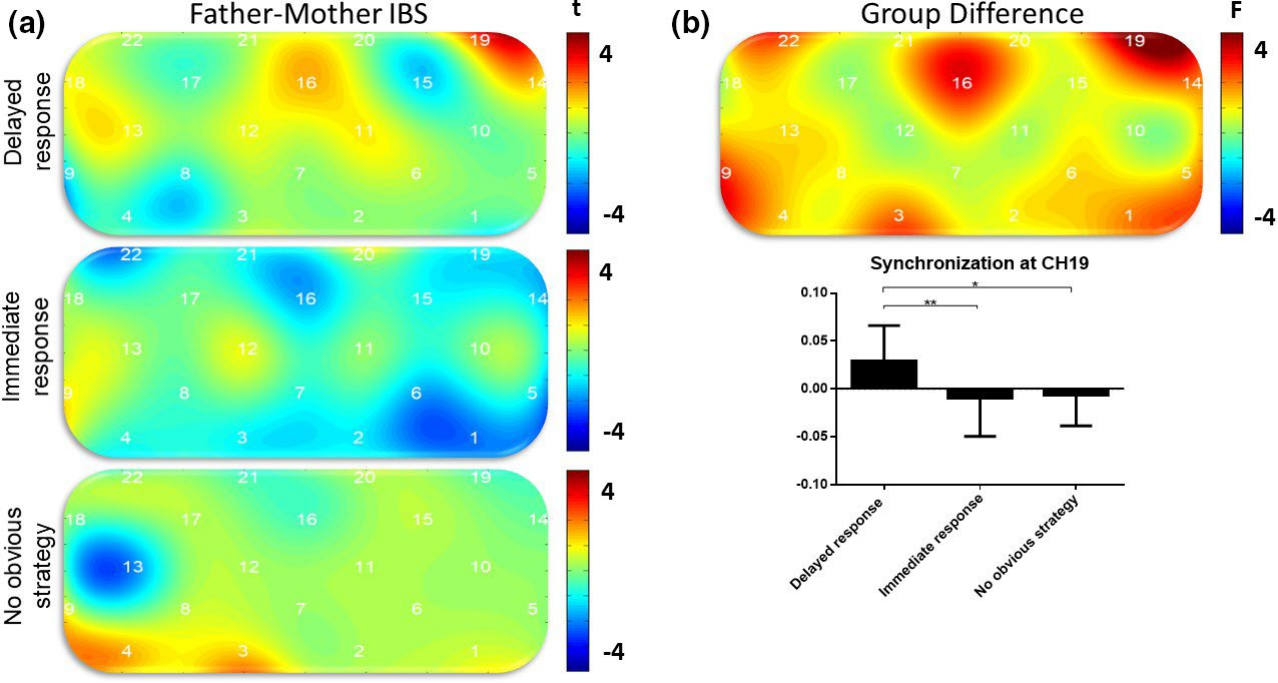
Value map of the IBS of different strategy groups. (a) One‐sample *t* test map of IBS. (b, upper) One‐way ANOVA results of IBS to identify group differences. (b, lower) The amplitude of synchronization at channel 19. Significant IBS at channel 19, after FDR correction, was only found in delayed response dyads. Synchronization in delayed response dyads is higher than that in other dyads.*p < .05, **p < .01, ***p < .001

Given the widespread conclusion that IBS can significantly predict task performance (Baker et al., 2016; Cui et al., 2012; Pan et al., 2017; Reindl et al., 2018; Wang, Zhang, et al., 2019; Wang, Han, et al., 2019), the series of one‐sample *t* tests and independent‐sample *t* tests we used were all one‐tailed. In the delayed response group, we found significant IBS in channel 19 (*t*(15) = 3.58, *p* = .0019), which passed FDR correction (*p* < .05); in the immediate response group, there was no channel where significant IBS occurred; in the no obvious strategy group, IBS was significant in channel 3 (*t*(9) = 2.41, *p* = .02) but did not pass FDR correction (Figure 6a). A series of one‐way ANOVAs revealed differences in IBS among different strategy groups in channel 19 (*F*(2,39) = 4.46, *p* = .02, $\eta_{p}^{2}$ = 0.235; see Figure 6b). Post hoc analysis showed that the IBS of channel 19 was significantly stronger in the delayed response group than in the immediate response group (*p* = .009) and the no obvious strategy group (*p* = .045). In addition, IBS in channel 19 was significantly correlated with partners’ CC (*r* = .38, *p* = .031) but not with task performance (WR), RTD, or threshold, which was consistent with our second hypothesis, namely that CC can significantly predict participants’ IBS.

In a GCA of oxy‐Hb signals in channel 19 of couples in the delayed response group, the GC values in both directions were significant (from males to females: *t*(16) = 4.03, *p* < .001; from females to males: *t*(16) = 5.24, *p* < .001), but there was no significant difference between the two directions (*t*(16) = −0.75, *p* = .47).

### 3.3 Scales

#### 3.3.1 Comparisons of relevant indicators between couples

Males’ parenting stress was significantly higher than females’ (*t*(35) = 2.30, *p* = .03), but there were no significant differences in the other two dimensions—parent–child dysfunctional interaction and difficult child—and in the total parenting stress scores. There were also no significant differences in the total score of DAS and the four subscales (marital satisfaction, marital harmony, marital cohesion, and emotional expression).

#### 3.3.2 Relationship between parental traits, task performance, and IBS

We have found that strategies decisively impact on performance in this cooperative task. So which participant traits affect their choice of cooperative strategies? On the one hand, the total score of females’ parenting stress was positively correlated with task performance (WR) (*r* = .39, *p* = .019); on the other hand, males’ parenting stress (*r* = .39, *p* = .019) and parent–child dysfunctional interaction (*r* = .389, *p* = .02) were also positively correlated with task performance (WR). These results seem to indicate an association between parenting stress and cooperation strategy choice, although for males the influence was mainly reflected in the two dimensions of parenting stress and parent–child dysfunctional interaction. According to the effects of different strategies, we coded delayed response strategy as “1,” immediate response strategy as “2,” and no obvious strategy as “3.” Then, we analyzed the correlations between related indicators and each couple's cooperation strategy and found that strategies negatively correlated with the scores of males’ parent–child dysfunctional interaction (*r* = −.49, *p* = .003) and the total scores of females’ parental stress (*r* = −.41, *p* = .013). Generally, the greater a couple's perceived parenting stress, the more likely they were to adopt the delayed response strategy. Couples’ strategy selection, task performance, and IBS were not related to the DAS total score or to any of the four DAS dimensions (marital satisfaction, marital harmony, marital cohesion, and emotional expression). We also found that males’ educational level was the only demographic variable significantly correlated with strategy selection (*r* = −.39, *p* = .02): specifically, the higher the male's educational level, the greater the likelihood of the couple adopting the delayed response strategy.

### 3.4 Subjective measurements

There was no significant difference between couples in the five subjective indicators measured at the end of the task. Correlations between WR, IBS, CC, and the subjective measurements are shown in Table 1.

**Table 1:** Correlations between WR, IBS, CC, and the subjective measurements

	Males					Females				
	Shared intention	Performance satisfaction	Cooperation degree	Concentration degree	Pleasantness	Shared intention	Performance satisfaction	Cooperation degree	Concentration degree	Pleasantness
WR	0.523**	0.503**	0.657**	0.214	0.136	0.21	0.236	0.48**	0.21	0.199
IBS	0.328	0.409*	0.520**	0.142	0.206	0.305	0.198	0.402	0.068	0.277
CC	0.64**	0.388*	0.64**	0.001	0.443**	0.497**	0.407*	0.521**	0.299	0.411*

Abbreviations: CC, cooperation coefficient; RTD, Response time difference; WR, winning ratio.

* *p* < .05.

** *p* < .01.

As Table 1 shows, the better the performance of couple's cooperation, the higher the five subjective indicators, even if some do not reach a significant level. The relationships between task performance and these subjective indicators may have a corresponding neurophysiological basis, because the stronger the synchronization between partners’ brains, the more obvious the trend of these correlations, especially for males’ performance satisfaction and cooperation degree. CC is significantly correlated with four subjective indicators of couples (the exception being concentration degree), suggesting that the cooperation level between partners determines their task performance and IBS, and further affects both partners’ subjective evaluations. It should also be noted that the impact of CC on these subjective indicators is more extensive than that of task performance (WR) itself.

## 4 DISCUSSION

In this study, two commonly used cooperative strategies, delayed response, and instant response, were examined in cooperative keystroke tasks using an fNIRS‐based hyperscanning technique. Compared with the immediate response strategy and no obvious strategy, the delayed response strategy produced better task performance and was more conducive to generating IBS in the right frontal cortex. The better task performance of the delayed response strategy group reflects the higher level of cooperation within dyads in this group, as measured by the cooperation coefficient (CC) we constructed. Furthermore, CC significantly predicted dyads’ task performance, IBS, and task‐related subjective measurements.

The average thresholds of dyads in the delayed response strategy group were significantly larger than those in the other two groups, but the RTD within these dyads was basically equal to the RTD of dyads in the other two groups. Hence, it is reasonable that the mean CC of the delayed response strategy group was significantly larger than that of the other two groups. For this reason, winning through the delayed response strategy may be described as a fluke as it results from enlarging the threshold, not reducing the RTD. However, it is very difficult for a delayed response dyad with longer RTs to keep their RTD basically equal to that of an immediate response dyad, since an immediate response is more instinctive and leads to shorter RTs. Therefore, if dyads can successfully execute a delayed response, then they deserve to win.

It should also be noted that IBS in channel 19 was only significantly correlated with CC (and not with RTD, threshold, or WR). This may further reveal the relationship between the cooperative task and IBS. In dyads adopting the delayed response strategy, whose delay time to keep behavioral synchronization (which averaged about 0.5 s in this study) was accompanied by a smaller RTD, IBS was more likely to trigger in the right frontal cortex. Previous studies have revealed that IBS requires both partners to engage in the same psychological activities for common goals (e.g., Baker et al., 2016; Cui et al., 2012; Pan et al., 2017; Reindl et al., 2018; Wang, Han, et al., 2019). Adopting the delayed response strategy can better unify the response modes of both partners. It enables both sides to react purposefully according to the established rhythm (estimated delay time after seeing the signal), triggers synchronized mentalization processes (predicting each other's keystrokes) and self‐control processes (controlling themselves to press keys after a certain delay), and enables their brains to become synchronized. In this study, the neurological activity of the right frontal cortices of dyad partners tended to be synchronized. This is consistent with previous studies of cooperation and interaction between humans related to the right frontal‐parietal cortex (Decety et al., 2004), and with the conclusion that time counting can trigger IBS (Funane et al., 2011; Mu, Guo, & Han, 2016). In conclusion, higher levels of cooperation are more likely to trigger IBS in the corresponding brain regions. There is a significant correlation between CC (rather than WR) and IBS, which indicates that if the cooperation level (or cooperation contribution) between the two sides is not sufficient, even if task performance is not bad, it cannot effectively trigger the synchronization of corresponding brain regions. In addition, high‐level or high‐contribution cooperation will bring both partners a higher level of emotional experience‐related task completion.

According to our videos and live recordings of the experiment, the couples in the immediate response group are basically divided into two situations. In one, they took immediate response to the task for granted: for example, one participant commented, “What strategies can we have besides that?” In the other, they considered the delayed response strategy but believed that since the immediate response is simple and direct, there is no need to delay. Therefore, using the immediate response strategy seems to be the instinctive choice of participants faced with this task. On this basis, it seems reasonable for Cui et al. (2012) to use formula 1 to set the threshold of response time. If a fixed value is used to replace the dynamic threshold, which changes with the change in response time, it is bound to make the task too difficult for some participants and too easy for others. This is because the difference in the fastest response time between individuals is stable. If dyad partners instinctively react to the signals instantly, those who react more closely will naturally achieve better results than those who react more differently. Therefore, compared with the delayed response group, participants in the immediate response group invested less in the task, and their task performance and IBS were relatively inferior.

The GCA results showed no significant difference, which was inconsistent with the previous finding of a significantly higher GC value from females to males than from males to females (Pan et al., 2017). It should be noted that the earlier study found that boyfriends took longer to respond than girlfriends, suggesting that males might deliberately make their button‐pressing movements stable later than their girlfriends, so as to maintain RTD within the threshold. This was consistent with the results of that study's GCA: women guide the key‐press response and brain activity of couples. Moreover, the steady lagging of males’ response relative to females’ indicates that males might dominate keystroke task‐based cooperation. However, in this study, there was no significant difference between couples in the delayed response strategy group and the GCA results showed that couples responded according to the strategies formed in the task; neither males nor females led the behaviors and IBS between them. What explains the differences between these two studies’ results? First, college lovers are still in the early stages of romantic relationships. Males in such relationships are more emotionally involved in the task than those participating in the experiment with a female friend or stranger. Driven by the motivation of continuing to develop this romantic relationship, they actively adjust their reaction patterns to adapt to the rhythm of their partners (Hoshi, 2007). However, the participants in this study have been married for more than six years. Males seemed to have no strong motivation to show their abilities to their partners. Second, according to the data analysis, couples’ parenting stress related to their cooperative strategies but not to their mutual adjustment level (DAS scores). This may mean that couples’ marital quality and status hardly affect either their strategy selection or their behavioral and neurological synchronization. On the contrary, the more parenting stress couples have, the stronger their emotional involvement in the task and the stronger their motivation to achieve better performance, leading to a greater likelihood of thinking of and adopting a more effective cooperative strategy: delayed response. Another potential factor affecting strategy selection is the male's educational level: compared to females, males’ strategy selection seems more closely associated with their education experience.

From the perspective of strategy selection, this study provides a new explanation for the performance of participants in the same or similar cooperative keystroke tasks and the generation of IBS, and proposes a reliable quantitative predictor of task performance and IBS—the cooperation coefficient—that helps deepen understanding of the cooperative keystroke task paradigm. However, given equipment limitations, it is currently impossible to monitor the neurological activity of two or more persons in the whole cerebral cortex simultaneously using fNIRS‐based hyperscanning. This study found significant IBS in couples’ right frontal cortex, whereas some studies that have employed the same task found IBS occurring in other brain regions. For instance, in the left frontal cortex, IBS was found to be sensitive to shared intentionality between dyad partners and correlated with the mutual prosocial inclination (e.g., Hu et al., 2017). IBS has also been found in the right temporal cortex, which has been implicated in social perception, action observation, and theory of mind (e.g., Baker et al., 2016). It seems that both these regions may relate to forming and implementing cooperation strategy. Therefore, follow‐up studies should test the possibility that IBS occurs in other brain regions (including but not limited to the two aforementioned) for married couples or other kinds of participants. It would also be interesting in future studies to inform participants of how the threshold is calculated, or use a fixed value as the threshold to determine win or loss on the task, and then analyze how participants’ strategies are affected. Moreover, follow‐up studies could examine the cooperation level in the same task between fathers and mothers and between children and their parents, measuring the related IBSs and the correlations among them.

## 5 CONCLUSION

In summary, in a cooperative keystroke task, a dyad adopting the delayed response strategy is likely to outperform dyads selecting the immediate response strategy and those with no obvious strategy, and also likely to have stronger IBS in the right frontal cortex. These findings suggest that the delayed response strategy may better unify dyad partners’ response modes, trigger synchronized psychological processes, and enable their brains to become synchronized. This study tested the efficiency of various cooperation strategies observed in such a task, providing a new perspective for future studies that may employ the same or a similar task paradigm.

## CONFLICT OF INTEREST

The authors declare no conflict of interests.

## AUTHORS’ CONTRIBUTIONS

Y.T., L.C., and F.L. designed the experiment. Y.T., X.L., and S.D. collected data. Y.T. and X.L. analyzed data. Y.T., X.L., C.W., M.C., S.D., X.D., Y.D., S.G., Y.F., L.C., and F.L. wrote the paper.

### Peer Review

The peer review history for this article is available at https://publons.com/publon/10.1002/brb3.1768.

## Data Availability

Datasets are available on request. The raw data and the data generated during analyses that support the findings of the study will be made available by the authors, without undue reservation, to any qualified researcher.
